# Public interest in retinal detachment in the United States: a Google Trends analysis

**DOI:** 10.1186/s40942-026-00870-x

**Published:** 2026-06-01

**Authors:** Thanaphat Seeboonruang, Muhammad Z. Chauhan, Qais A. Dihan, Ahmed F. Shakarchi, Jamie L. Surgent-Nahay, Ahmed B. Sallam

**Affiliations:** 1 Department of Ophthalmology, Harvey and Bernice Jones Eye Institute, University of Arkansas for Medical Sciences, 4301 W Markham Street #523, Little Rock, AR 72205 USA; 2 Department of Ophthalmology, Faculty of Medicine, Ain Shams University, Cairo, Egypt

**Keywords:** Public interest, Retinal detachment, Google Trends

## Abstract

**Background:**

Rhegmatogenous retinal detachment (RD) is a vision-threatening condition requiring timely diagnosis and treatment, yet population-level public interest and information-seeking behavior is difficult to assess using traditional utilization data alone. This study evaluated temporal and geographic patterns of public interest in retinal detachment in the United States and contextualized this interest relative to retina specialist availability.

**Methods:**

In this retrospective observational study, we queried Google Trends for RD–related search terms in the United States from 2008 to 2025. Google Trends reports relative search volume (RSV) on a normalized scale from 0 to 100. We extracted annual and state-level values using a composite of diagnostic and surgical RD terms. We obtained state-level counts of vitreoretinal surgeons from the American Society of Retina Specialists database and calculated retina surgeon density per 100,000 population using 2025 U.S. Census estimates. We also calculated the Search-to-Surgeon Index as an exploratory ratio of state-level relative search volume to retina surgeon density. We evaluated associations between RSV, Search-to-Surgeon Index, population age structure, and time using the using the Mann–Kendall trend test with Sen’s slope estimator and the Spearman correlation test.

**Results:**

From 2008 to 2025, retinal detachment–related relative search volume increased by 178%, with a significant temporal increasing trend (τ = 0.745, *P* < 0.001). Relative search volume declined sharply in April 2020. In 2025, state-level relative search volume ranged from 61 to 100 (median, 84). States with a higher proportion of residents aged 50 years and older demonstrated higher relative search volume (ρ = 0.56, *P* < 0.001). Retina surgeon density varied more than tenfold across states, ranging from 0.17 to 2.09 per 100,000 residents. Search-to-Surgeon index values ranged from 40 to 406 (median, 155), with the highest values observed in states with limited retina surgeon availability. Population age distribution was not significantly associated with Search-to-Surgeon index (ρ = -0.06, *P* > 0.10).

**Conclusions:**

Public interest in retinal detachment has increased substantially over time and varies widely across U.S. states. Integrating Google Trends data with retina surgeon distribution highlights geographic mismatches between public interest and specialist availability. This exploratory framework may help identify regions with disproportionate public concern relative to local workforce capacity.

## Introduction

Rhegmatogenous retinal detachment (RD) is a vision-threatening condition that often requires urgent surgical intervention to prevent permanent visual loss [1]. The incidence of RD has increased over time, driven by population aging, rising prevalence of myopia, and prior ocular surgery, particularly cataract surgery, which is a well-established risk factor [2, 3].

Public interest and information-seeking behavior regarding RD surgery is challenging to quantify using data from claims or surgical volume alone, as these metrics reflect service utilization rather than public awareness. Google Trends offer an option for quantifying real-time public interest in RD and RD-related surgery [4]. This tool has been used in multiple ophthalmic contexts [5], but has not been applied to retina surgery.

In this study, we used Google Trends to examine temporal and geographic variation in public interest related to RD across the United States (U.S.) and contextualized this interest relative to retina surgeon availability.

## Methods

We conducted a retrospective observational ecological infodemiology study using publicly available Google Trends data (accessed on January 10, 2026) in conjunction with U.S. Census data to assess temporal and geographic patterns of public interest in retinal detachment in the United States. Given the use of publicly available, nonidentifiable aggregate data which did not involve direct patient contact or intervention, individual informed consent was not required. This study was exempted by the Institutional Review Board of the University of Arkansas for Medical Sciences. This study was conducted in accordance with the principles outlined in the Declaration of Helsinki.

### Google Trends query strategy

We queried Google Trends for searches originating in the United States between 2008 and 2025. Google Trends reports relative search volume (RSV) on a normalized scale from 0 to 100, with 100 representing peak popularity for a given search term within the selected time and geography. We used the following composite search terms combined using the Google Trends ‘+’ operator to capture public interest in RD: “Retinal Detachment,” “Detachment of the retina,” “Pneumatic Retinopexy,” “Scleral Buckle.” We purposefully excluded “vitrectomy” because it is a non-specific surgical term frequently performed for conditions unrelated to RD. We extracted annual and state-level RSV values from these terms. We applied the Mann–Kendall trend test with Sen’s slope estimator to assess both the direction and magnitude of temporal trends in RSV over the study period.

### Retina surgeon density

We obtained state-level counts of vitreoretinal surgeons from the American Society of Retina Specialists (ASRS) member database [6]. Because ASRS represents the largest professional organization of retina specialists in the U.S., surgeons performing higher volumes of vitreoretinal surgery are more likely to be members. We calculated retina surgeon density for each state by dividing the number of ASRS members by the 2025 U.S. Census Bureau state population estimates and multiplying by 100,000 [7].

### Search-to-surgeon index

We calculated a Search-to-Surgeon Index (SSI) to contextualize public interest relative to specialist availability. We defined SSI as the ratio of state-level RSV to retina surgeon density (SSI = state-level RSV ÷ retina surgeon density). Because RSV is a normalized, unitless scale rather than an absolute search count, the SSI is utilized strictly as an exploratory relative metric to rank geographic disparities rather than to quantify absolute clinical volume or demand. Higher SSI value indicated greater discrepancy between public interest and surgeon availability.

### Population age distribution

Because Google Trends does not provide demographic data on users’ age, we independently obtained state-level population age distributions from the 2025 U.S. Census Bureau. We used the proportion of residents aged ≥ 50 years as a proxy for populations at higher risk for symptomatic RD. We evaluated the correlation between age distribution, RSV, and SSI using Spearman correlation test.

## Results

### State-level retina specialist availability and population age structure

We identified 1,992 U.S.-based ASRS members. Retina surgeon density ranged from 0.17 to 2.09 per 100,000 residents (mean, 0.58). Delaware (2.09), Maryland (1.04), and Vermont (0.93) had the highest densities, whereas Wyoming (0.17), Alaska (0.27), and New Hampshire (0.28) had the lowest. Census data showed the highest proportions of residents aged ≥ 50 years in Maine, New Hampshire, Vermont, and West Virginia, and the lowest proportions in Utah, Texas, Alaska, and North Dakota.

### Search trends

**Fig. 1:**
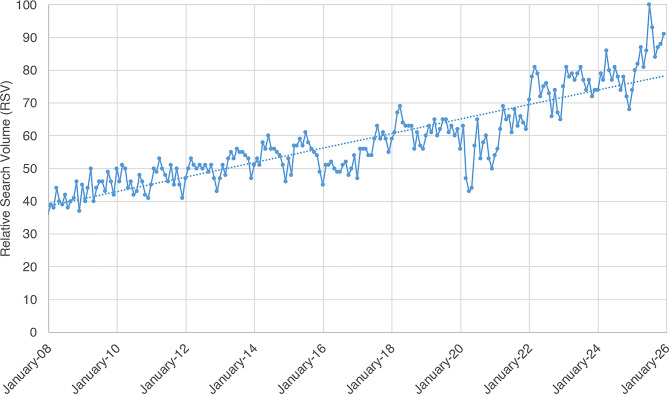
Time-series of RSV for composite retinal detachment–related search terms from January 2008 to December 2025. RSV = relative search volume

**Fig. 2:**
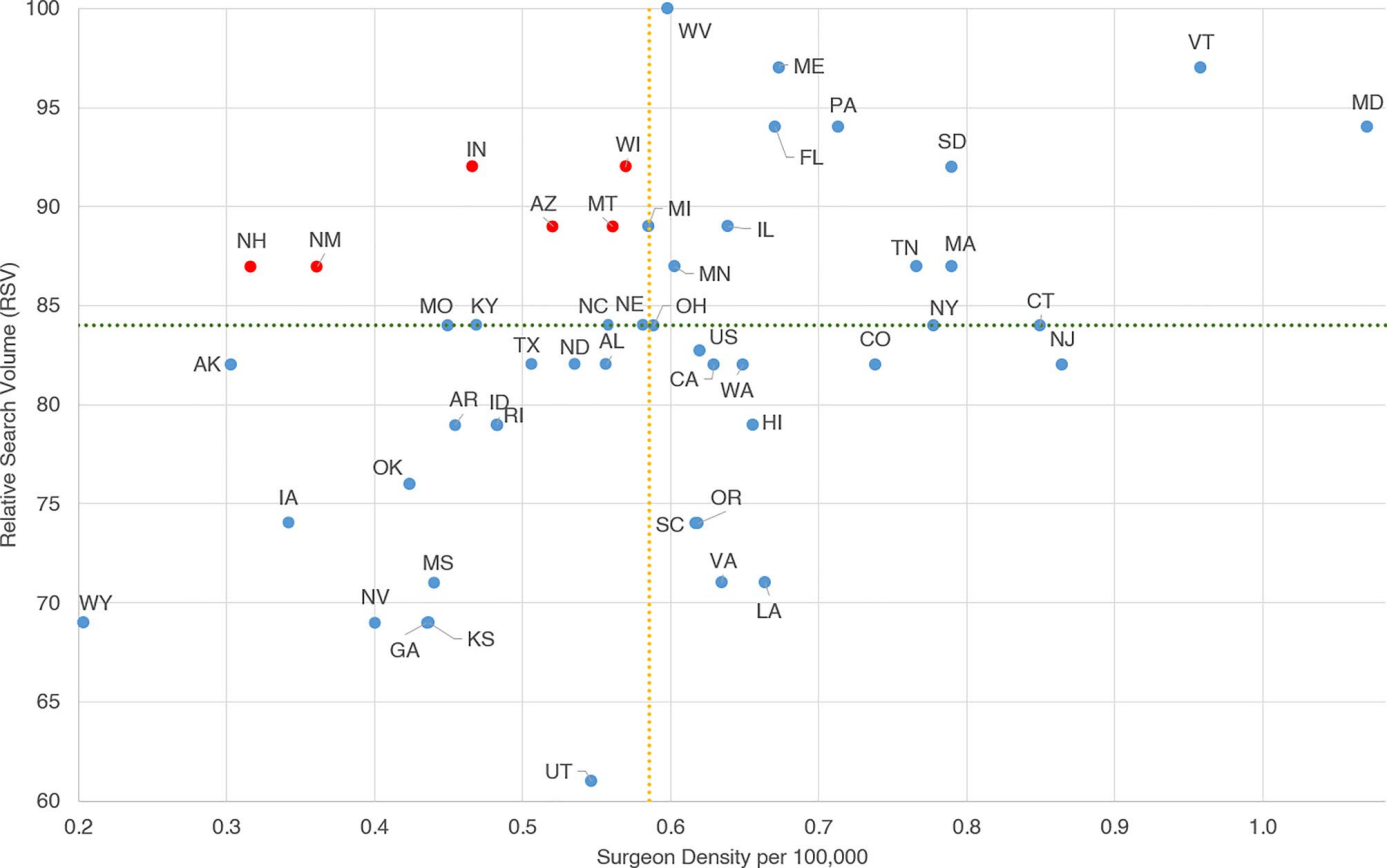
Relationship between RSV for composite retinal detachment–related search terms and surgeon density per 100,000 people in 2025. Green dash: median RSV. Orange dash: median surgeon density. Red dot: state with RSV above the median and surgeon density below the median. We used two-letter state abbreviations. RSV = relative search volume. Delaware (RSV, 40.2; surgeon density, 2.09) was excluded from the figure to enhance interpretability

From 2008 to 2025, RSV increased by 178%. Temporal trend analysis using the Mann–Kendall test demonstrated a strong positive monotonic association between time and RSV values (Kendall’s τ = 0.745, *P* < 0.0001) and Sen’s slope estimator indicated a median increase of 0.182 RSV units per month (95% CI: [0.169, 0.194]). The minimum RSV of 36 occurred in January 2008. There was a sharp decline in April 2020 (Fig. 1). In 2025, RSV ranged from 61 to 100 (median 84), with the highest values in West Virginia (100), Maine (97), and Vermont (97), and the lowest in Utah (61), Wyoming (69), Kansas (69), Nevada (69), and Georgia (69) (Fig. 2). There was a moderate positive correlation between the proportion of residents aged ≥ 50 years and RSV (ρ = 0.56, *P* < 0.001).

### Search-to-surgeon index

**Fig. 3:**
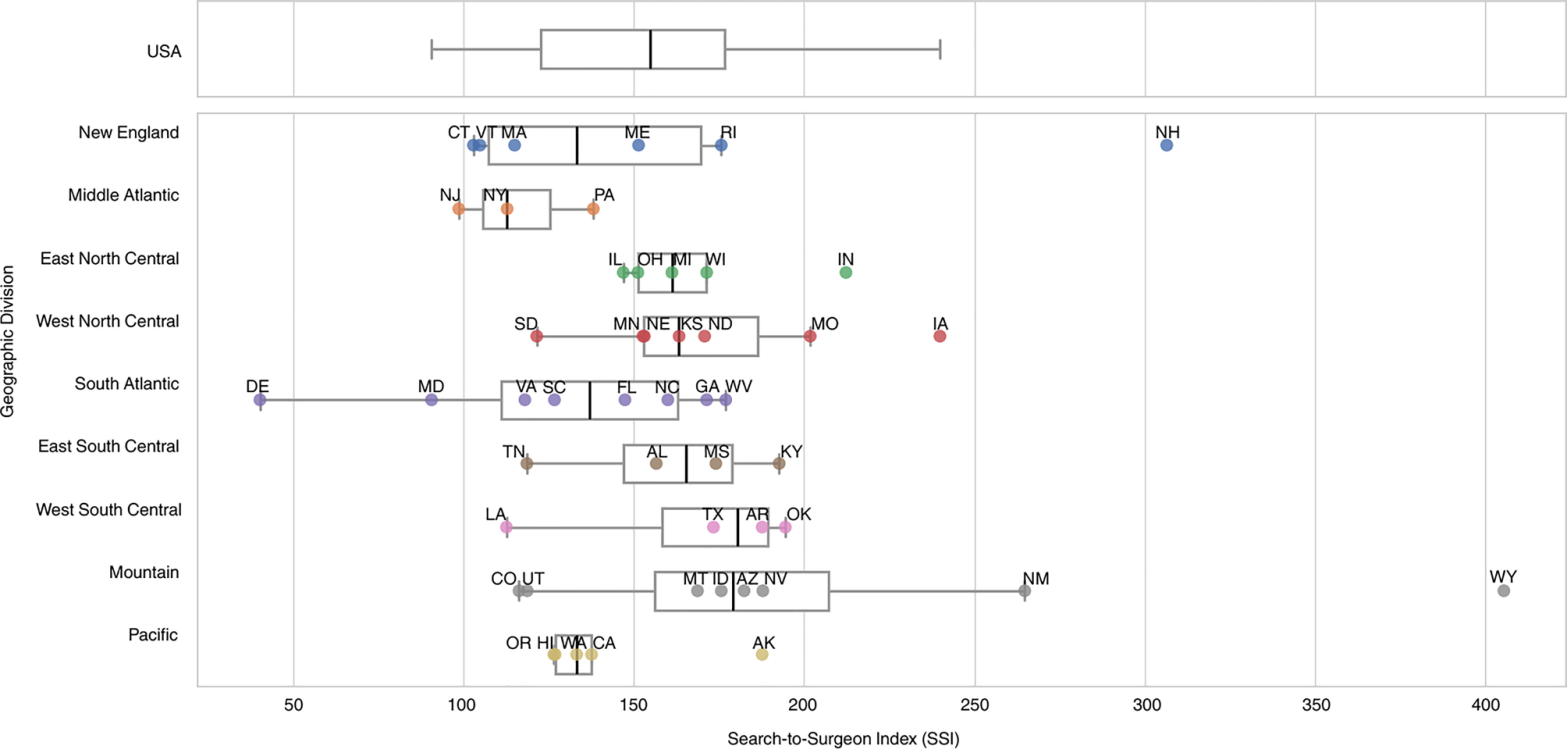
SSI for retinal detachment across the United States (U.S.) by Census Bureau geographic division. Overall, U.S. SSI is represented by the topmost boxplot. We used two-letter state abbreviations. SSI = Search-to-Surgeon index

The SSI ranged from 40 to 406 (median 155). Wyoming (406), New Hampshire (307), and Alaska (304) had the highest SSIs, whereas Delaware (40), Maryland (91), and New Jersey (99) had the lowest (Fig. 3). There was no correlation between population age distribution and SSI (ρ = -0.06, *P* > 0.10).

## Discussion

Over the past two decades, public search interest in RD has increased by nearly 2-fold. This trend likely reflects increasing information-seeking behavior associated with population aging, higher prevalence of myopia, and prior ocular surgery, all of which have been identified as important risk factors for RD [9]. In parallel, advances in vitreoretinal surgical techniques and improved outcomes may have increased public awareness and willingness to seek information about RD symptoms and treatment options [8].

Increased RD-related search activity may also reflect broader trends in online health information–seeking behavior [10]. Despite the rapid emergence of artificial intelligence–based tools, Google remains the dominant platform for online information retrieval and health-related searches. Global Google search volume has increased substantially over time, from approximately 60–70 billion searches per month in 2008 to more than 200 billion searches per month by 2014, reflecting a marked expansion in search-based information consumption [10, 11].

We noted a sharp decline in RSV occurred in early 2020, coinciding with the Coronavirus Disease 2019 pandemic. This finding mirrors reported reductions in hospital admissions and delayed presentations for RD during this period, likely due to reduced access to care and patient hesitancy to seek emergency services [12].

Geographically, RSV varied widely across states, with higher values observed in states with a greater proportion of residents aged 50 years and older. The moderate positive correlation between age structure and RSV supports the use of RD-related search interest as a proxy for population-level awareness consistent with established epidemiologic patterns of RD [8]. Retina specialist density varied more than tenfold across states. States such as Wyoming, New Hampshire, and Alaska demonstrated the highest SSI value, reflecting high search interest despite limited specialist availability. Conversely, states with higher retina surgeon density exhibited lower SSI. Given the time-sensitive nature of RD management, these geographic mismatches may have important implications for regional healthcare planning, particularly in rural or underserved regions [13].

Our study has several limitations. First, due to the ecological nature of this study, state-level RSV cannot be used to infer individual-level care-seeking behavior. Google Trends reports relative rather than absolute search volume and does not provide demographic information, including age, sex, or race or ethnicity, or user intent. Therefore, we cannot differentiate searches made by patients experiencing symptoms from those made by medical students, clinicians, or the public reacting to media. We therefore derived age distributions independently from U.S. Census data rather than inferring demographics from search behavior. While Google Trends normalizes RSV as a proportion of total searches to account for overall internet volume, the baseline growth in global internet access and digital health literacy over our 17-year study period may still partially confound the observed temporal increases. Not all individuals seek medical information online, and media attention may transiently influence search patterns. Our query strategy focused on formal clinical and surgical terms. The exclusion of colloquial phrases such as “detached retina” or “retinal detachment symptoms” may have omitted a subset of relevant patient searches. Although ASRS membership likely captures most surgeons performing retinal detachment surgery, the database may underrepresent non-member surgeons, particularly those in private practice. ASRS density is a crude proxy that does not account for an individual surgeon’s clinical capacity, actual surgical volume, cross-state geographic catchment areas, or availability for time-sensitive emergency call.

In conclusion, this analysis demonstrates substantial geographic variability in public interest in retinal detachment across the U.S. Integrating Google Trends data with retina surgeon distribution provides an exploratory framework for evaluating relative interest and potential geographic disparities in vitreoretinal care. Refining workforce estimates and identifying additional drivers of public interest may further clarify regional disparities.

## Data Availability

All data analyzed in this study were obtained from publicly accessible sources, including Google Trends and the American Society of Retina Specialists database. Requests for additional information regarding the data underlying this article should be directed to the corresponding author.
